# Effects of Heat Stress on Production Performance and Protein Metabolism of Skeletal Muscle in Meat Rabbits

**DOI:** 10.3390/ani15172560

**Published:** 2025-08-31

**Authors:** Gongyan Liu, Ce Liu, Haitao Sun, Liya Bai, Liping Yang, Yin Zhang, Shuxia Gao

**Affiliations:** Key Laboratory of Livestock and Poultry Multiomics of MARA, Institute of Animal Husbandry and Veterinary Medicine, Shandong Academy of Agricultural Sciences, Jinan 251000, China; gongyanliu@foxmail.com (G.L.); liuceshiyan@163.com (C.L.); wwww8888@163.com (H.S.); bailiya_2005@163.com (L.B.); yanglp682@163.com (L.Y.); insaas@foxmail.com (Y.Z.)

**Keywords:** heat stress, rabbits, protein metabolism, carcass yield, meat quality

## Abstract

The purpose of this experiment was to study the effects of heat stress on the performance and protein metabolism of skeletal muscle in meat rabbits. A total of 160 New Zealand White rabbits aged 80 days with mean initial body weights of 2359 ± 200 g were randomly divided into a control group and a heat stress group. The experiment duration was 20 days. Heat stress treatment reduced the growth performance and slaughter performance of the rabbits (*p* < 0.05) and increased muscle yellowness (b*, *p* < 0.05). In addition, heat stress treatment increased the concentrations of leptin, cholesterol, high-density lipoprotein (HDL) cholesterol, and low-density lipoprotein (LDL) cholesterol in serum (*p* < 0.05), and decreased the serum total protein and immunoglobulin (IgG, IgM, and IgA) contents of rabbits. Under the criteria fold-change ≥ 1.20 or ≤0.84 and *p*-value ≤ 0.05, 7 up-regulated proteins and 122 down-regulated proteins were screened. A gene ontology (GO) enrichment analysis of the differentially expressed proteins was performed. The most enriched specific GO terms among the differential proteins were response to stress, extracellular region, and protein binding in the biological process (BP), cellular component (CC), and molecular function (MF) categories, respectively, and the most enriched pathway was the PI3K/Akt signalling pathway. In conclusion, heat stress could reduce the carcass yield of meat rabbits, change the physical characteristics of the skeletal muscle, and influence protein metabolism by changing blood indices, potentially through the PI3K/Akt signalling pathway.

## Simple Summary

In modern animal husbandry production, rabbits are readily affected by heat stress in summer. A critical issue in rabbit production is to determine the mechanism by which heat stress prevents muscle growth. In this study, we found that heat stress reduced the carcass yield of meat rabbits, changed the physical characteristics of the skeletal muscle, and influenced protein metabolism by changing blood indices, potentially through the PI3K/Akt signalling pathway.

## 1. Introduction

In modern animal husbandry production, livestock and poultry are readily affected by many abiotic stress factors that negatively impact animal performance, especially heat stress in summer [1]. The entire body of the meat rabbit is coated, the sweat gland function is underdeveloped, body temperature regulation is poor, and the animal is readily subject to heat stress, all of which affect its production performance and product quality [2,3]. One crucial issue in rabbit production is to elucidate the mechanism by which heat stress prevents muscle growth. Meat is the most important product in animal production, and the reduced growth caused by heat stress is an impediment to overall animal performance and farm profitability [4,5]. Short-term heat stress may also lead to a decline in performance and weight loss in pigs [6]. Skeletal muscle catabolism increases during heat stress and leads to an increase in plasma markers of muscle catabolism in several species [7,8,9,10]. Li et al. [11] reported that heat stress has no direct effect on cow insulin and leptin secretion. However, heat stress leads to lower dry matter feed intake, which increases insulin and leptin concentrations on top of the same dry matter feed intake. Due to changes in muscle metabolism, effective muscle growth and function may be countered. Increasing evidence shows that heat stress changes intracellular metabolism, causing increased glycolysis and incomplete oxidative phosphorylation [12]. With the rapid development of genomics, proteomics, and other technologies, the use of tandem mass tag (TMT)-based proteomics has become a powerful tool to explore the biological processes of animal growth and development and is widely used in the research on the regulatory mechanisms of animal production traits [13]. Zheng et al. [14] used a TMT-based proteomic technique to study differentially expressed proteins in bovine mammary glands at peak lactation and late lactation. A total of 179 differentially expressed proteins were screened, of which 14 were associated with lactation performance and mammary gland morphology. Wang et al. [15] studied differentially expressed proteins during the development of skeletal muscle in sheep embryos using a TMT-labelling proteomic technique, as well as identified 1316 differentially expressed proteins and further analyzed the functions of these proteins. However, the application of TMT-based proteomics technology in rabbits is rare. Moreover, in the Fujian province of China, people like to use rabbits weighing more than 3 kg to make soup. Based on the above requirements, in this experimental study, we chose to use purebred New Zealand rabbits as experimental animals, and the purebred New Zealand rabbits can reach a weight of 3 kg at the age of 100 days, at which time the slaughter achieves a better yield. Therefore, the effect of heat stress on the growth performance, slaughter performance, and meat quality of New Zealand White rabbits was investigated from 80 days to 100 days, and the protein metabolism of skeletal muscle was evaluated using a TMT proteomics approach.

## 2. Materials and Methods

### 2.1. Experimental Design

In this experiment, 160 New Zealand White rabbits aged 80 days with a mean initial body weight of 2359 ± 200 g and in good health were purchased by the Qingdao Kangda Rabbit Industry Development Co., Ltd., Qingdao, China. The rabbits were randomly divided into two groups, namely a control group and a heat stress treatment group, with 10 replicates in each group and eight rabbits in each replicate. The experimental animals were fed in two completely identical closed rabbit houses. In the control group, the air temperature was maintained at 25–28 °C with adequate ventilation, and the relative humidity was 50–60%. For the heat stress treatment group, the air temperature was maintained above 32 °C, and a humidifier maintained the relative humidity at 70–80%. The treatment period was 20 days. The rabbits were fed the same diet (the feed ingredients and nutritional levels are shown in Table 1), and feed and water were provided ad libitum. During the experiment, the temperature and humidity near the door, window, and in the centre of the house in the feeding room of the control group and the heat stress group were measured with a digital thermometer every day, and the daily average temperature and humidity were calculated. The temperature–humidity index (THI) formula proposed by Marai et al. [16] was used to calculate the environmental THI: THI = db − [(0.31 − 0.31RH)/(db − 14.4)], where db and RH are the thermometer value (°C) and relative humidity (%), respectively.

### 2.2. Sample Collection and Preparation

At the end of the experiment (100 days of age), ten experimental rabbits in each treatment (one rabbit whose weight was close to the average weight of this repetition was selected) were selected for sample collection. A 10 mL blood sample was collected from the heart and then centrifuged at a centrifugal force of 1500 g for 10 min. The isolated serum samples were stored at −80 °C for the determination of serum biochemical indices. In addition, the longissimus thoracis et lumborum (LTL) muscles from both sides of each carcass were collected to determine their physical properties. A sample (1 g) of the LTL muscle was placed in a freezing tube and stored in liquid nitrogen. Samples of four rabbits randomly selected from the control group and the treatment group were selected for inspection, and a TMT-based quantitative proteomic analysis was conducted.

### 2.3. Determination of Indicators and Methods

#### 2.3.1. Growth Performance

At the beginning and end of the experiment, the weight of each rabbit was measured, and the average daily gain was calculated. The average daily feed intake was calculated by dividing the total feed intake of each repetition by the total number of days of the experiment and the number of test rabbits. The feed–gain ratio was calculated as feed intake/weight gain.

#### 2.3.2. Slaughter Performance

Twelve hours prior to slaughter, the rabbits were fasted and weighed to determine the preslaughter body weight. The slaughter procedure and carcass analysis were performed as described by Blasco and Ouhayoun [17]. Before slaughter, the animals were stunned by electric shock and then slaughtered by bloodletting. After bleeding, the pelts, paws, and full gastrointestinal tract were removed, and the semiclean carcass weight was the carcass weight after removing the head at the first cervical vertebra, removing the trachea and esophagus, and retaining the heart, liver, and kidney. The full clean carcass weight is the semiclean carcass weight after removing the heart, liver, and kidney. The head, heart, liver, and kidney were also weighed. The semiclean slaughter ratio and full clean slaughter ratio were calculated by dividing their weights by the live weight before slaughter.

#### 2.3.3. Blood Indices

Commercial radioactive immune assay kits supplied by Tianjin Jiuding Company (Tianjin, China) were used to analyze insulin, glucagon, and leptin contents in serum, and radioactivity was determined in DFM-96 10 tubes with a radioactive immune gamma counter (Hefei Zhongcheng Electromechanical Technology Development Co., Ltd., Hefei, China). Serum glucose, cholesterol, triglyceride, high-density lipoprotein (HDL) cholesterol, and low-density lipoprotein (LDL) cholesterol were determined with a sequential multiple analyzer (Hitachi 7020, Tokyo, Japan) following the manual of commercial protocols (Wako, Japan). The IgG, IgM, and IgA concentrations were measured by immunoturbidimetry using enzyme-linked immunosorbent assay kits (CEA544Rb, CEA543Rb, and SEA641Rb, respectively; Cloud-Clone Corp., Wuhan, China).

#### 2.3.4. Meat Physical Characteristics

The pHu values (pH measured at 24 h postmortem), muscle colour (L*, a*, and b*), drip loss ratio, cooking loss ratio, and shear force were measured following a previous report [18].

#### 2.3.5. Identification and Bioinformatic Analysis of Protein

After retrieving the original data from the database, blank values were removed. Based on credible proteins, the differentially expressed proteins were screened based on different screening conditions. A gene ontology (GO) analysis of the identified differentially expressed proteins was performed with Blast2GO software(Version 4.1.9). The attributes of these proteins were described in relation to the biological process, molecular function, and cellular component. The identified differential proteins were uploaded to the UniProt database (http://www.uniprot.org/uploadlists/ (accessed on 28 August 2025)), exported as a fasta file, and the enriched Kyoto Encyclopedia of Genes and Genomes (KEGG) pathways were ascertained to determine the most important metabolic pathways involving the differentially expressed proteins.

### 2.4. Statistical Analysis

The data were analyzed by analysis of variance (ANOVA) followed by Duncan’s multiple range test. The general linear model (GLM) procedure of SAS 9.4 statistical software (SAS Institute Inc., Cary, NC, USA) was used. The data are expressed as the mean ± standard deviation, and *p* < 0.05 was considered to be significant.

## 3. Results and Analysis

### 3.1. Temperature—Humidity Index

**Figure 1:**
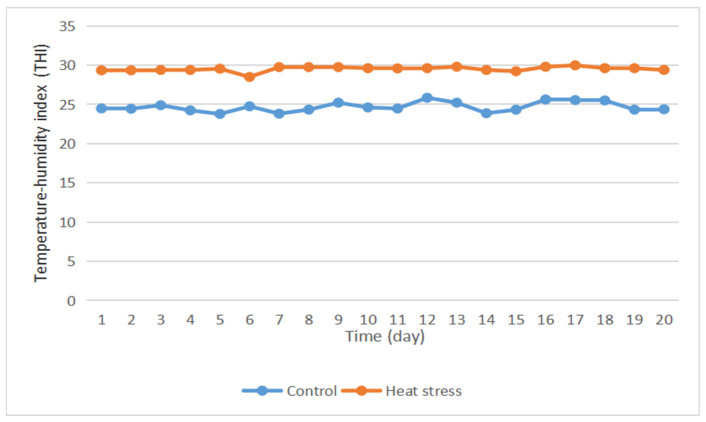
Diurnal variation curve of temperature and humidity index during the experiment.

- Heat stress in rabbits can be divided into four grades: no heat stress at THI < 27.8; moderate heat stress at 27.8 < THI < 28.9; severe heat stress at 28.9 < THI < 30.0; and especially severe heat stress at THI > 30. The average daily THI of the control group was lower than 27.8 (Figure 1), indicating that the experimental rabbits in this group were in a state of no heat stress throughout the experiment. The average daily THI of the heat stress group was greater than 28.9, and thus, the rabbits were in a state of heat stress.

### 3.2. Growth Performance

Heat stress treatment reduced the average daily feed intake and average daily gain of the rabbits (*p* < 0.05), but there was no observed difference in the feed–gain ratio (*p* > 0.05; Table 2).

### 3.3. Slaughter Performance

The preslaughter body weight, semiclean carcass weight, full clean carcass weight, semiclean slaughter ratio, full clean slaughter ratio, and liver weight of the experimental group treated with heat stress were lower than those of the control group (*p* < 0.05; Table 3). No differences in head weight, heart weight, and kidney weight were observed in this experiment (*p* > 0.05).

### 3.4. Blood Indices

Heat stress treatment increased the concentrations of leptin, cholesterol, HDL, and LDL in serum (*p* < 0.05), and decreased the serum total protein and immunoglobulin (IgG, IgM, and IgA) contents of the rabbits (Table 4). No effects on serum insulin, glucagon, and glucose contents were observed (*p* > 0.05).

### 3.5. Physical Characteristics of the LTL Meat

Heat stress changed the muscle colour and increased muscle yellowness (b*, *p* < 0.05). However, no differences in the pHu value, shear force, drip loss ratio, and cooking loss ratio were observed (*p* > 0.05; Table 5).

### 3.6. Protein Metabolism of Skeletal Muscle

#### 3.6.1. Data Quality Control and Identification

The mass spectrometry data were searched against the uniprot-oryctolagus-cuniculus-filtered-organism-Oryctolagus-cuniculus protein database. The obtained fasta file contained 23,058 sequences. To improve the quality of the analysis results and reduce the false positive rate, Proteome Discoverer 2.2 software was used to filter the search results. Peptide spectrum matches (PSMs) with more than 99% confidence were trusted PSMs, and proteins containing at least one unique peptide segment were trusted proteins. Only the trusted peptides and proteins were retained, and those with a false discovery rate > 1% were removed. In this study, there were a total of 324,622 spectra, 79,888 matched spectra, 18,941 identified peptides, 2463 identified proteins, and 2460 quantifiable proteins.

#### 3.6.2. Protein Function Annotation

**Figure 2:**
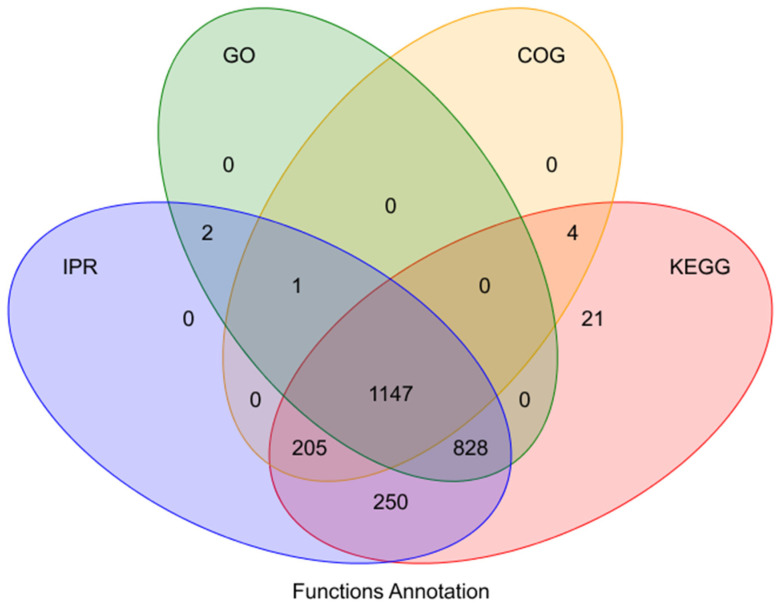
Results of function annotation.

To explore the functional characteristics of the differential proteins, the identified proteins were annotated based on information in the GO, KEGG, COG, and IPR databases. In total, 1147 proteins were annotated from these databases (Figure 2).

#### 3.6.3. Protein Quantitative Analysis

**Figure 3:**
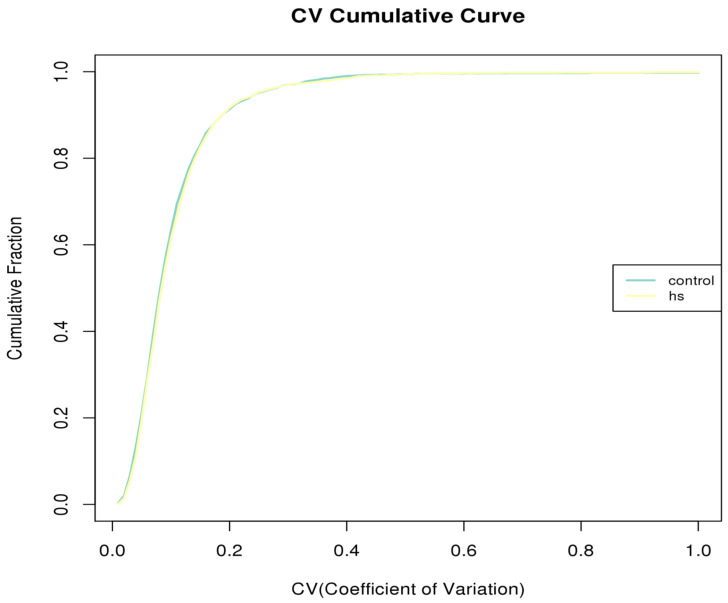
Coefficient of variation analysis, hs: heat stress.

The coefficient of variation (CV), which is the ratio of the standard deviation to the mean, was used to measure the degree of variation in each observed value of a sample, which reflects the dispersion degree of the data, and thus, is a measure of repeatability. The smaller the CV value, the better the repeatability (Figure 3).

#### 3.6.4. Differential Protein Analysis

**Figure 4:**
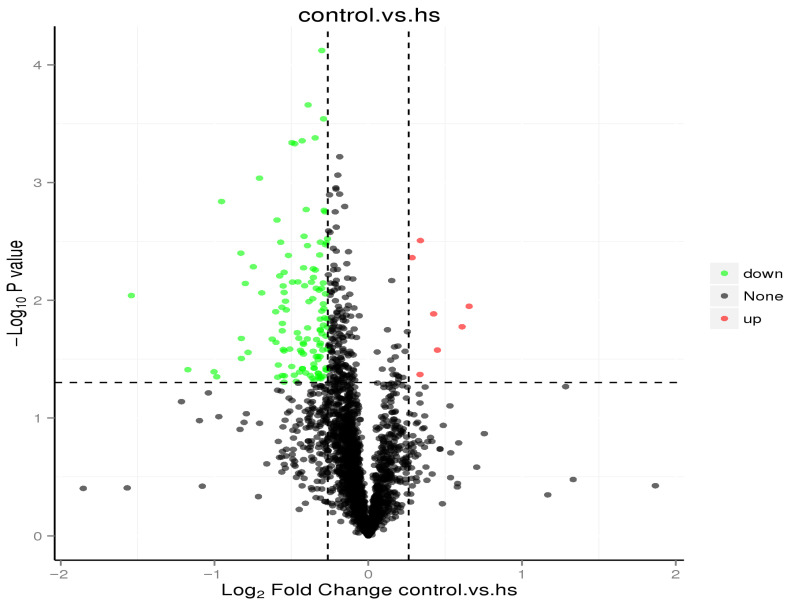
Volcano map of differential proteins, hs: heat stress.

Applying the criteria of fold-change (FC) ≥ 1.20 or ≤0.84 and *p*-value ≤ 0.05, 7 up-regulated proteins and 122 down-regulated proteins were screened (Table 6). With FC ≥ 1.30 or ≤0.77 and *p*-value ≤ 0.05, 4 up-regulated proteins and 64 down-regulated proteins were screened. With FC ≥ 1.50 or ≤0.67 and *p*-value ≤ 0.05, 2 up-regulated proteins (G1SUJ3, Histone H2A, and B7NZF9, Nucleophosmin 1 isoform 1, predicted) and 19 down-regulated proteins were screened (Table 7). Finally, with FC ≥ 2.00 or ≤0.50 and *p*-value ≤ 0.05, no up-regulated proteins and three down-regulated proteins (P01870, Ig gamma chain C region; A0A1Y1B8B3 and A0A1Y1BG72, IgG heavy chain VDJ region, fragment) were screened. For each protein multiple of difference, the log2-transformed FC and log10-transformed absolute *p*-value were plotted to generate a volcano map (Figure 4).

#### 3.6.5. Enrichment Analysis

**Figure 5:**
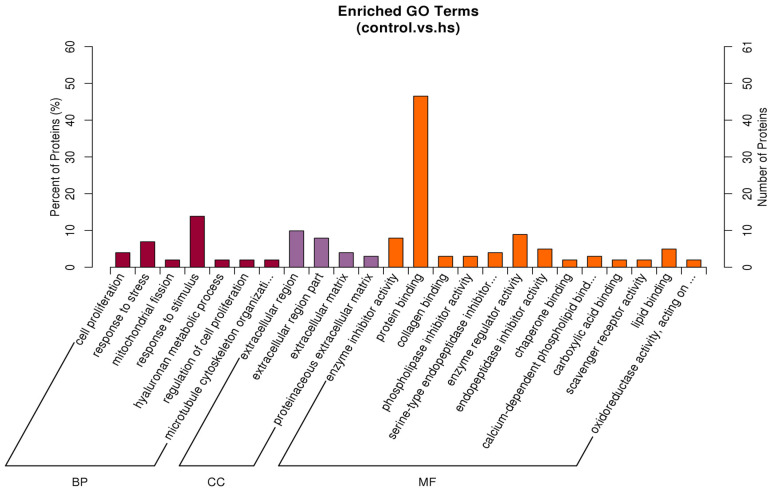
The histogram of GO enrichment for three categories, each of which shows up to 20 kinds (*p*-value ≤ 0.05). The percentage of ordinate represents x/n in the table. BP: biological process; CC: cellular component; MF: molecular function, hs: heat stress.

**Figure 6:**
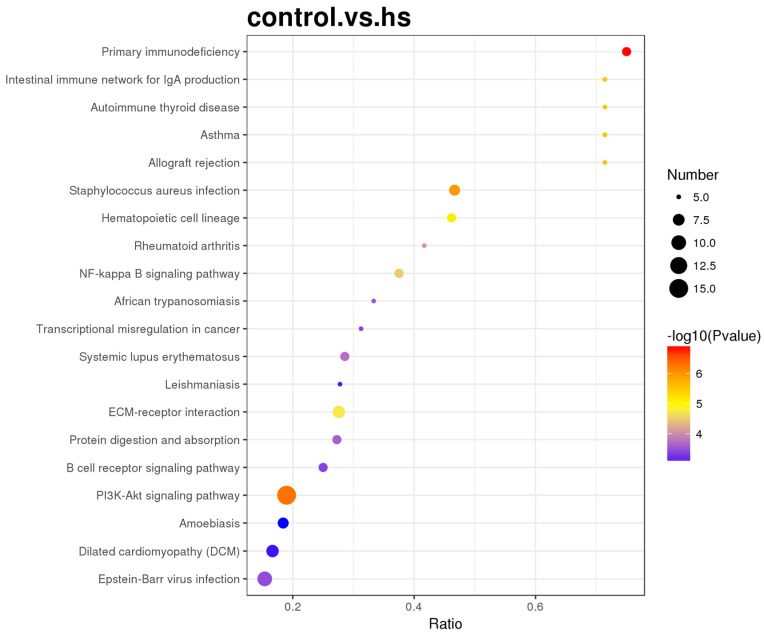
The bubble diagram of KEGG enrichment, hs: heat stress.

The most enriched GO terms among the differential proteins were response to stress, extracellular region, and protein binding in the biological process, cellular component, and molecular function categories, respectively (Figure 5). The target genes were identified in the KEGG database to determine the pathways involving the differential proteins. The most enriched pathway was the PI3K/Akt signalling pathway (Figure 6).

## 4. Discussion

Growth involves a series of complex metabolic events, which are controlled by heredity and environment. Heat stress can reduce the productivity of almost all livestock breeds [19]. As warm-blooded animals, rabbits use physical, morphological, biochemical, and behavioural processes to regulate their body’s heat input and output to maintain a constant body temperature [20]. The temperature of a rabbit’s thermal neutral zone is approximately 18–21 °C [21]. Therefore, under exposure to high ambient temperature, the body temperature of a rabbit will be out of balance, which has adverse effects on growth and reproduction traits [22,23,24]. Previous studies have reported a reduction in rabbit feed consumption under heat stress [25] because high ambient temperature stimulates the peripheral heat receptors to transmit inhibitory nerve impulses to the appetite centre of the hypothalamus. In this study, total and daily weight gains in growing rabbits were suppressed. The reduction in daily gain was due to a decrease in rabbit feed intake (215.31 vs. 185.76 g/d; Table 2), which might have led to decreased protein biosynthesis and less fat deposition [26,27].

Owing to changes in muscle metabolism, effective muscle growth and function may be countered [28]. Increasing evidence shows that heat stress changes intracellular metabolism and that these changes indicate an increase in glycolysis and incomplete oxidative phosphorylation [5]. Lactic acid production and pyruvate kinase activity in the muscle of broilers increase under chronic heat stress, which provides evidence of increased glycolytic ability [29]. Similarly, when exercising at high temperatures, the plasma lactic acid concentration will increase [30]. With an increase in temperature, the decrease in serum total protein seems to be due to the dilution of serum total protein caused by the increase in water consumption, and/or it may be due to the increase in protein utilization and amino acid transamination in heat-stressed rabbits. Heat stress will affect the lipid metabolism of broilers, and the high cholesterol and triglyceride contents in the blood reflect enhanced lipid catabolism [31]. Leptin is a hormone secreted by adipose tissue and involved in promoting fat breakdown and utilization, and its content in serum is proportional to the size of the animal’s adipose tissue [32,33]. Leptin acts on receptors located in the central nervous system to regulate the behaviour and metabolism of organisms [34]. Leptin regulates the energy balance and weight of organisms through a negative feedback mechanism [35]. Leptin has the effect of suppressing appetite, increasing protein degradation, and inhibiting fat synthesis, affecting many physiological systems and metabolic pathways of the body [36]. The HDL receptor mediates the selective uptake of cholesterol [37]. Rinaldo and Le [38] showed that heat stress may reduce the activity of glucose-6-phosphate dehydrogenase, thus reducing the activity of reduced nicotinamide adenine dinucleotide dehydrogenase and inhibiting energy metabolism. In addition, we observed that heat stress reduced the content of immunoglobulin in the blood, which may lead to a decrease in animal immunity and an increase in mortality during a hot summer.

Heat stress also affected the amount and type of volatile substances in muscle, which affected muscle odour [39], dietary supplementations of vitamin C, organic selenium, betaine, and the effect of pomegranate peel on alleviating the effect of heat stress on growing rabbits [40]. Our findings show that heat stress increased the expression of the proteins G1SUJ3 (Histone H2A) and B7NZF9 (Nucleophosmin 1 isoform 1, predicted), and decreased the expression of the proteins P01870 (Ig gamma chain C region), A0A1Y1B8B3, and A0A1Y1BG72 (IgG heavy chain VDJ region, fragment). Moreover, the most enriched specific GO terms for the differential proteins were response to stress, extracellular region, and protein binding in the biological process, cellular component, and molecular function categories (Figure 5). As the central link in the insulin pathway, the PI3K/AKT axis regulates hepatic glycogen synthesis, gluconeogenesis, and lipid synthesis [41,42]. In addition, the PI3K/AKT axis regulates lipogenesis by inhibiting a sterol regulatory element-binding transcription factor (SREBP-1c), subsequently increasing hepatic LDL receptor protein expression [43,44].

## 5. Conclusions

Heat stress disrupts the growth and carcass yield of growing meat rabbits, alters some physical characteristics of the skeletal muscle, and influences protein metabolism and lipid metabolism by changing blood indices. These responses may be mediated through the PI3K/Akt signalling pathway.

## Figures and Tables

**Table 1:** Composition and nutritional level of the experimental diets (dry matter basis, %).

Raw Material Composition	Content	Nutrients ^(2)^	Content
Corn	17.0	Digestible energy (MJ/kg)	10.28
Soybean meal	10.0	Crude protein	16.20
Wheat middling	8.0	Crude fibre	17.47
Wheat bran	12.0	Ash	11.75
Corn germ meal	10.0	Crude fat	2.79
Peanut straw powder	25.0	Lysine	0.60
Sunflower meal	8.0	Methionine	0.27
Soybean straw powder	7.0	Calcium	0.97
Premix ^(1)^	3.0	Total phosphorus	0.43
Total	100.0		

^(1)^ Premix provided the following per kg of diets: calcium hydrogen phosphate 1.5 g; salt 5 g; lysine 1.5 g; methionine 1.5 g; Fe 70 mg; Zn 60 mg; Cu 20 mg; Mn 30 mg; Se 0.05 mg; vitamin A 8000 IU; vitamin D3 1500 IU; vitamin E 45 mg; vitamin K 3.0 mg; thiamine 5 mg; riboflavin 10 mg; pantothenic acid 20 mg; niacin 35 mg; choline 400 mg. ^(2)^ Except for digestible energy, all other nutritional levels are measured values.

**Table 2:** Effects of heat stress on growth performance of meat rabbits.

Items	Control	Heat Stress	Root Mean Square Error	*p*-Value
Initial body weight (g)	2392.0 ± 68.7	2326.0 ± 56.1	198.3543	0.4665
Average daily gain (g/d)	62.90 ± 3.20 ^a^	51.70 ± 4.73 ^b^	10.8282	0.0201
Average daily feed intake (g/d)	215.31 ± 13.32 ^a^	185.76 ± 11.36 ^b^	20.6241	0.0362
Feed–gain ratio	4.07 ± 0.81	3.99 ± 0.67	2.3614	0.9369

The data are expressed as the mean ± standard deviation; n = 10 per group. ^a,b^ different letters denote significance (*p* < 0.05).

**Table 3:** Effects of heat stress on slaughter performance of meat rabbits.

Items	Control	Heat Stress	Root Mean Square Error	*p*-Value
Preslaughter body weight (g)	3650.0 ± 102.5 ^a^	3360.0 ± 96.7 ^b^	222.9132	0.0426
Semiclean carcass weight (g)	1964.0 ± 76.6 ^a^	1934.0 ± 78.6 ^b^	153.5178	0.0288
Full clean carcass weight (g)	1887.0 ± 74.1 ^a^	1865.4 ± 78.4 ^b^	152.5478	0.0440
Semiclean slaughter ratio (%)	57.72 ± 1.17 ^a^	54.05 ± 0.50 ^b^	2.1116	0.0173
Full clean slaughter ratio (%)	51.93 ± 1.13 ^a^	49.67 ± 0.63 ^b^	2.7265	0.0167
Head weight (g)	123.8 ± 3.1	125.7 ± 2.9	9.4331	0.6578
Heart weight (g)	6.1 ± 0.5	5.0 ± 0.2	1.1762	0.0510
Liver weight (g)	58.4 ± 2.1 ^a^	52.1 ± 1.6 ^b^	5.9503	0.0293
Kidney weight (g)	12.5 ± 0.5	11.5 ± 0.9	2.3688	0.3577

The data are expressed as the mean ± standard deviation; n = 10 per group. ^a,b^ different letters denote significance (*p* < 0.05).

**Table 4:** Effects of heat stress on blood indices of meat rabbits.

Items	Control	Heat Stress	Root Mean Square Error	*p*-Value
**Serum hormone indices**				
Insulin (μIU/mL)	5.12 ± 1.00	6.06 ± 0.93	3.0460	0.4974
Glucagon (μIU/mL)	270.33 ± 6.55	263.12 ± 8.77	24.4779	0.5186
Leptin (ng/mL)	13.71 ± 0.79 ^b^	21.09 ± 0.79 ^a^	2.5105	0.0001
**Serum biochemical indices (mmol/L)**				
Glucose	2.62 ± 0.44	2.73 ± 0.27	1.1491	0.6526
Total protein	60.28 ± 1.94 ^a^	50.08 ± 1.78 ^b^	5.8900	0.0041
Cholesterol	1.08 ± 0.16 ^b^	1.71 ± 0.21 ^a^	0.5882	0.0285
Triglyceride	3.20 ± 0.92	3.68 ± 0.31	2.1696	0.6227
High-density lipoprotein (HDL)	0.53 ± 0.09 ^b^	0.96 ± 0.13 ^a^	0.3571	0.0160
Low-density lipoprotein (LDL)	0.51 ± 0.08 ^b^	0.89 ± 0.08 ^a^	0.2497	0.0034
**Serum immune indices**				
Immunoglobulin G (IgG, μg/mL)	8.11 ± 0.14 ^a^	6.87 ± 0.28 ^b^	0.7141	0.0011
Immunoglobulin M (IgM, ng/mL)	687.66 ± 11.73 ^a^	654.52 ± 9.29 ^b^	33.4621	0.0399
Immunoglobulin A (IgA, ng/mL)	124.97 ± 0.87 ^a^	118.09 ± 2.86 ^b^	6.6971	0.0338

The data are expressed as the mean ± standard deviation; n = 10 per group. ^a,b^ different letters denote significance (*p* < 0.05).

**Table 5:** Effects of different ambient temperatures on meat quality of meat rabbits.

Items	Control	Heat Stress	Root Mean Square Error	*p*-Value
pHu value	6.17 ± 0.06	6.10 ± 0.05	0.1785	0.4475
Lightness (L*)	49.45 ± 1.06	50.66 ± 0.83	5.2146	0.3712
Redness (a*)	11.26 ± 0.66	11.04 ± 0.75	3.8607	0.8277
Yellowness (b*)	6.79 ± 0.38 ^b^	8.02 ± 0.35 ^a^	2.0105	0.0214
Shear force (N)	32.35 ± 2.53	31.22 ± 2.10	7.3544	0.7354
Drip loss ratio (%)	2.60 ± 0.32	3.45 ± 0.35	1.0508	0.0876
Cooking loss ratio (%)	36.74 ± 0.67	35.14 ± 0.65	2.0777	0.1022

The data are expressed as the mean ± standard deviation; n = 10 per group. ^a,b^ different letters denote significance (*p* < 0.05).

**Table 6:** The quantity of differential proteins.

Compared Samples	Quantifiable Protein	Regulated Type	Fold-Change > 1.2 or ≤0.84	Fold-Change > 1.3 or ≤0.77	Fold-Change > 1.5 or ≤0.67	Fold-Change > 2.0 or ≤0.50
Control vs. Heat Stress (hs)	2460	Up	7	4	2	0
		Down	122	64	19	3

**Table 7:** The information on differential proteins (fold-change > 1.5 or ≤0.67).

Protein	Description	Gene	Fold-Change	*p*-Value	log2 Fold-Change	Regulated Type
G1SUJ3	Histone H2A	H2AFV	1.5757	0.0113	0.6560	up
B7NZF9	Nucleophosmin 1 isoform 1 (predicted)	NPM1	1.5266	0.0168	0.6104	up
P01870	Ig gamma chain C region	Unmatched	0.3437	0.0091	−1.5406	down
A0A1Y1B8B3	IgG heavy chain VDJ region (fragment)	Unmatched	0.4434	0.0389	−1.1732	down
A0A1Y1BG72	IgG heavy chain VDJ region (fragment)	Unmatched	0.4989	0.0405	−1.0031	down
G1T4P1	Fibrinogen C-terminal domain-containing protein	ANGPTL7	0.5052	0.0447	−0.9852	down
A0A1Y1B9K3	IgM light chain (fragment)	Unmatched	0.5162	0.0014	−0.9539	down
G1TRK9	Ig-like domain-containing protein	Unmatched	0.5630	0.0040	−0.8287	down
G1TMM0	Inter-alpha-trypsin inhibitor heavy chain H3	ITIH3	0.5643	0.0211	−0.8255	down
G1SM64	Uncharacterized protein	ITIH2	0.5644	0.0312	−0.8252	down
G1TVZ5	Ig-like domain-containing protein	Unmatched	0.5744	0.0072	−0.7998	down
G1T5B1	LRRNT domain-containing protein	KERA	0.5817	0.0277	−0.7816	down
G1U2T3	Uncharacterized protein	STIMATE	0.5957	0.0052	−0.7473	down
A0A1Y1B9M1	IgM light chain (fragment)	Unmatched	0.6125	0.0009	−0.7072	down
A0A1Y1B8Z0	IgG light chain (fragment)	Unmatched	0.6187	0.0087	−0.6927	down
G1SEK8	Uncharacterized protein	FETUB	0.6488	0.0214	−0.6242	down
P01687	Ig kappa chain V region BS-5	Unmatched	0.6588	0.0125	−0.6020	down
G1T3Z1	C-type lectin domain-containing protein	MBL2	0.6597	0.0228	−0.6001	down
G1THZ6	Uncharacterized protein	Unmatched	0.6627	0.0021	−0.5936	down
A0A1Y1BDX9	IgG light chain (fragment)	Unmatched	0.6649	0.0450	−0.5888	down
P01697	Ig kappa chain V region AH80-5	Unmatched	0.6666	0.0355	−0.5851	down

## Data Availability

Datasets obtained and/or analyzed during the current study are available from the corresponding author upon reasonable request.
